# Supplementary material for: Mechanistic Understanding Enables the Rational Design of Salicylanilide Combination Therapies for Gram-Negative Infections
Source: mBio. 2020 Sep 15;11(5):e02068-20. doi: 10.1128/mBio.02068-20 (PMC7492738; doi:10.1128/mBio.02068-20)
Supplement: TABLE S1 [file mBio.02068-20-st001.pdf]

Table S1 | *E. coli* strains utilized in this study

E. coli strain	Relevant characteristics and references	Niclosamide MIC (μg.mL^{-1})
Wild-type BW25113	Δ*(araD-araB)567*, Δ*lacZ4787*(::rrnB-3), λ^{-}, *rph-1*, Δ*(rhaD-rhaB)568*, *hsdR514* [16738554]	>32
Δ*acrA*	Deletion of AcrA membrane fusion protein of resistance-nodulation-division family (1)	>32
Δ*acrB*	Deletion of AcrB transporter of resistance-nodulation-division family (1)	>32
Δ*acrZ*	Deletion of AcrZ accessory protein of resistance-nodulation-division family (1)	>32
Δ*emrA*	Deletion of EmrA membrane fusion protein of major facilitator superfamily (1)	>32
Δ*emrB*	Deletion of EmrB transporter of major facilitator superfamily (1)	>32
Δ*macA*	Deletion of MacA membrane fusion protein of ATP-binding cassette family (1)	>32
Δ*macB*	Deletion of MacB transporter of ATP-binding cassette family (1)	>32
Δ*tolC*	Deletion of TolC outer membrane factor, interacts with multiple efflux families (1)	0.5
Δ*mdtP*	Deletion of MdtP outer membrane factor, interacts with multiple efflux families (1)	>32
Δ7NR	Deletion of seven azo- and nitro-reductase enzymes (AzoR, MdaB, NemA, NfsA, NfsB, YcaK, YieF) from *E. coli* K-12 W3110 (2)	>32
Δ7NR*tolC*	Δ7NR with additional deletion of the TolC outer membrane factor (2)	0.016

(1) Baba T, Ara T, Hasegawa M, Takai Y, Okumura Y, Baba M, Datsenko KA, Tomita M, Wanner BL, Mori H. 2006. Construction of Escherichia coli K-12 in-frame, single-gene knockout mutants: the Keio collection. Mol Syst Biol 2:2006.0008.

(2) Copp JN, Williams EM, Rich MH, Patterson AV, Smaill JB, Ackerley DF. 2014. Toward a high-throughput screening platform for directed evolution of enzymes that activate genotoxic prodrugs. Protein Eng Des Sel **27**:399–403.
