# Supplementary material for: Mechanistic Understanding Enables the Rational Design of Salicylanilide Combination Therapies for Gram-Negative Infections
Source: mBio. 2020 Sep 15;11(5):e02068-20. doi: 10.1128/mBio.02068-20 (PMC7492738; doi:10.1128/mBio.02068-20)
Supplement: TABLE S2 [file mBio.02068-20-st002.pdf]

Table S2 | The assessment of antibiotic compounds administered alone or in combination with oxyclozanide

Antibiotic	Compound class	Target	BW25113 MIC[a] (μg/mL)	BW25113 ZIP score[b]	BW25113 FICI[c]	Δ*tolC* MIC[a] (μg/mL)	Δ*tolC* ZIP score[b]	Δ*tolC* FICI[c]
Chloramphenicol	Amphenicol	Protein synthesis	32	5.6	0.38	2	1.2	0.75
Tetracycline	Tetracycline	Protein synthesis	2	9.0	0.25	0.5	-0.8	0.75
Gentamicin	Aminoglycoside	Protein synthesis	0.25	-6.3	9	0.5	-8.5	8.5
Fosfomycin	Phosphonic acid	Cell wall biosynthesis	32	-0.6	1	16	-3.6	2
Cefotaxime	Cephalosporin	Cell wall biosynthesis	0.064	10.8	0.38	0.016	-4.8	3
Meropenem	Carbapenem	Cell wall biosynthesis	0.128	5.7	0.38	0.128	-5.0	2.5
Rifampicin	Ansamycin	RNA synthesis	32	1.7	1	4	-2.2	2
Metronidazole	Nitroimidazole	DNA	1024	0.4	1	1024	5.0	1.25
Nitrofurantoin	Nitrofuran	DNA/multiple	8	8.5	0.75	2	3.8	0.5
Vancomycin	Glycopeptide	Cell wall biosynthesis	256	-0.3	2	256	-4.1	1
Trimethoprim	Pyrimidine inhibitor	Folate synthesis	4	2.9	0.38	0.25	2.2	0.75
Ciprofloxacin	Quinolone	DNA synthesis	0.032	6.1	0.75	0.032	-3.0	2

[a] MIC analyses were performed in M9 minimal media

[b] ZIP scores were calculated from checkerboard assays with oxyclozanide in M9 minimal media

[c] The fractional inhibitory concentration index (FICI) was calculated from checkerboard assays with oxyclozanide in M9 minimal media
