# Supplementary figures and images for: Mechanistic Understanding Enables the Rational Design of Salicylanilide Combination Therapies for Gram-Negative Infections
Source: mBio. 2020 Sep 15;11(5):e02068-20. doi: 10.1128/mBio.02068-20 (PMC7492738; doi:10.1128/mBio.02068-20)

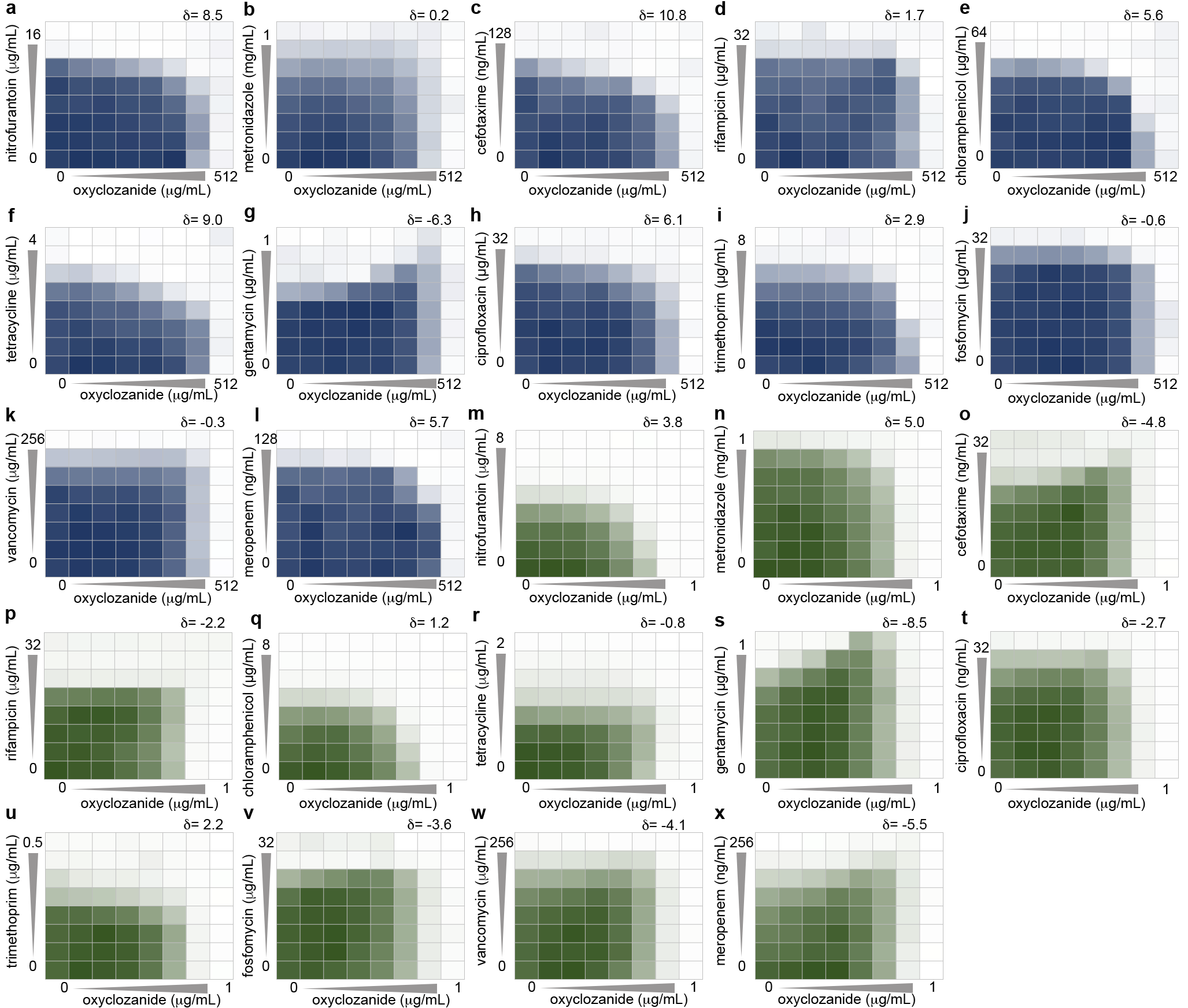

Supplement: FIG S4 [file mBio.02068-20-sf004.tif]
