# Supplementary material for: Mechanistic Understanding Enables the Rational Design of Salicylanilide Combination Therapies for Gram-Negative Infections
Source: mBio. 2020 Sep 15;11(5):e02068-20. doi: 10.1128/mBio.02068-20 (PMC7492738; doi:10.1128/mBio.02068-20)
Supplement: TABLE S3 [file mBio.02068-20-st003.pdf]

Table S3 | Clinical strains utilized in this study

Strain	Colistin MIC (µg/mL)	Relevant characteristics	Reference or source
Pseudomonas aeruginosa			
P. aeruginosa LESB58	4	Liverpool Epidemic Strain isolate; colistin resistant	(1)
P. aeruginosa NZRM4034	1	Ceftazidime/piperacillin resistant isolate	ESR resources
Acinetobacter baumannii			
A. baumannii Ab5075	0.25	Virulent wound isolate	(2)
A. baumannii NZRM3289	1	Urine isolate (ATCC19606)	ESR resources
A. baumannii C4	37.5	Colistin-resistant isolate; phosphatidylethanolamine addition to lipid A	(3)
Klebsiella pneumoniae			
K. pneumoniae KPLN49	0.5	Vancouver General Hospital Isolate	(4)
K. pneumoniae NZRM4387	1	Extended spectrum ß-lactamase urine isolate	ESR resources
Enterobacter cloacae			
E. cloacae 218R1	0.25	Class C ß-lactamase overproducing strain	(5)
Escherichia coli			
E. coli E38 (serotype O78:H^-)	0.25	Human peritoneum isolate	BEI Resources
E. coli NZRM4403	0.5	ß-lactamase overproducing human blood isolate	ESR resources
E. coli NCTC13846	3	Colistin resistant isolate; plasmid-borne mcr-1	(6)

References

(1) Cheng K, Smyth RL, Govan JR, Doherty C, Winstanley C, *et al*. 1996. Spread of β-lactam-resistant *Pseudomonas aeruginosa* in a cystic fibrosis clinic. Lancet 348:639-642

(2) Jacobs AC, Thompson MG, Black CC, Kessler JL, Clark LP, *et al.* 2014. AB5075, a highly virulent isolate of *Acinetobacter baumannii*, as a model strain for the evaluation of pathogenesis and antimicrobial treatments. mBio 5:e01076-14

(3) Arroyo LA, Herrara CM, Fernandez L, Hankins JV, Trent MS *et al*. 2011. The pmrCAB operon mediates polymyxin resistance in *Acinetobacter baumannii* ATCC17978 and clinical isolates through phosphoethanolamine modification of lipid A. Antimicrob Agents Chemother 55:3732-3752

(4) Behroozian S, Svensson SL, Davies J. 2016. Kisameet clay exhibits potent antibacterial activity against the ESKAPE pathogens. mBio 7:e01842-15

(5) Marchou B, Bellido F, Charnas R, Lucain C, Pechere JC. 1987. Contribution of β-lactamase hydrolysis and outer membrane permeability to ceftriaxone resistance in *Enterobacter cloacae*. Antimicrob Agents Chemother 32:1589095

(6) Akhoundsadegh N, Belanger CR, Hancock REW. 2019. Outer Membrane Interaction Kinetics of New Polymyxin B Analogs in Gram-Negative Bacilli. Antimicrob Agents Chemother 63:277.
